# Regenerative Therapeutic Potential of Adipose Stromal Cells in Early Stage Diabetic Retinopathy

**DOI:** 10.1371/journal.pone.0084671

**Published:** 2014-01-09

**Authors:** Gangaraju Rajashekhar, Ahmed Ramadan, Chandrika Abburi, Breedge Callaghan, Dmitry O. Traktuev, Carmella Evans-Molina, Raj Maturi, Alon Harris, Timothy S. Kern, Keith L. March

**Affiliations:** 1 Indiana Center for Vascular Biology & Medicine, Indiana University School of Medicine, Indianapolis, Indiana, United States of America; 2 Eugene and Marilyn Glick Eye Institute, Indiana University School of Medicine, Indianapolis, Indiana, United States of America; 3 Vascular and Cardiac Center for Adult Stem Cell Therapy, Indiana University School of Medicine, Indianapolis, Indiana, United States of America; 4 VA Center for Regenerative Medicine, Indiana University School of Medicine, Indianapolis, Indiana, United States of America; 5 Department of Ophthalmology, Indiana University School of Medicine, Indianapolis, Indiana, United States of America; 6 Department of Cellular & Integrative Physiology, Indiana University School of Medicine, Indianapolis, Indiana, United States of America; 7 Department of Biochemistry and Molecular Biology, Indiana University School of Medicine, Indianapolis, Indiana, United States of America; 8 Department of Medicine, Indiana University School of Medicine, Indianapolis, Indiana, United States of America; 9 Herman B Wells Center for Pediatric Research, Indiana University School of Medicine, Indianapolis, Indiana, United States of America; 10 Midwest Eye Institute, Indianapolis, Indiana, United States of America; 11 Departments of Medicine and Ophthalmology, Case Western Reserve University, Cleveland, Ohio, United States of America; Children's Hospital Boston, United States of America

## Abstract

Diabetic retinopathy (DR) is the leading cause of blindness in working-age adults. Early stage DR involves inflammation, vascular leakage, apoptosis of vascular cells and neurodegeneration. In this study, we hypothesized that cells derived from the stromal fraction of adipose tissue (ASC) could therapeutically rescue early stage DR features. Streptozotocin (STZ) induced diabetic athymic nude rats received single intravitreal injection of human ASC into one eye and saline into the other eye. Two months post onset of diabetes, administration of ASC significantly improved “b” wave amplitude (as measured by electroretinogram) within 1–3 weeks of injection compared to saline treated diabetic eyes. Subsequently, retinal histopathological evaluation revealed a significant decrease in vascular leakage and apoptotic cells around the retinal vessels in the diabetic eyes that received ASC compared to the eyes that received saline injection. In addition, molecular analyses have shown down-regulation in inflammatory gene expression in diabetic retina that received ASC compared to eyes that received saline. Interestingly, ASC were found to be localized near retinal vessels at higher densities than seen in age matched non-diabetic retina that received ASC. *In vitro*, ASC displayed sustained proliferation and decreased apoptosis under hyperglycemic stress. In addition, ASC in co-culture with retinal endothelial cells enhance endothelial survival and collaborate to form vascular networks. Taken together, our findings suggest that ASC are able to rescue the neural retina from hyperglycemia-induced degeneration, resulting in importantly improved visual function. Our pre-clinical studies support the translational development of adipose stem cell-based therapy for DR to address both retinal capillary and neurodegeneration.

## Introduction

Diabetic retinopathy (DR) is the most common vascular complication in patients with long-standing diabetes, and is the leading cause of blindness in working-age adults. The estimated prevalence in the USA is 5.4% (∼7.7 million) [1]. Future projections suggest that DR will become a larger public health problem, with an increase in the obese population as well as an increase in the prevalence of diabetes [2]. In the early stages of DR (non-proliferative DR; NPDR), clinically significant microvascular changes have been reported which presumably develop concomitant with pericyte loss, basement membrane thickening, and endothelial dysfunction involving loss of barrier integrity [3]. These changes cause the leakage of exudative material into the macula resulting in visual loss. It is becoming increasingly clear that neuronal cells of the retina also are affected by diabetes, resulting in visual dysfunction and even degeneration of some neuronal cells [4], [5]. Diabetes causes metabolic and physiologic abnormalities in the retina, and these changes suggest a role for inflammation in the development of DR. Using pharmacologic inhibitors or genetically modified animals, development of at least the early stages of DR, especially occlusion and degeneration of retinal capillaries has been documented[6], [7]. More recently, proinflamatory proteins released from neutrophils and monocytes have been shown to be critical in this process [8]. The development of such degenerate capillaries is preceded by the loss of pericytes (specialized perivascular cells), which are believed to provide a nourishing, anti-inflammatory and anti-angiogenic environment for endothelial cells [9]. Furthermore, the formation of the blood-retinal barrier (BRB) is dependent on the interaction of the vascular endothelial cells with both glial cells and pericytes [10]. Therefore loss of pericytes has been suggested to play a key role in the development of DR [11].

Current strategies for the therapeutic management of DR include symptomatic treatments such as laser photocoagulation, intravitreal triamcinolone and intravitreal injection of VEGF neutralizing agents (e.g., Ranibizumab), but these therapies achieve only limited success [12]. Furthermore, there are no treatments for reversing the severe ischemic changes that occur with more advanced disease and/or macular ischemia, the primary cause of reduced visual function/blindness. The Diabetes Control and Complications Trial, one of the largest clinical trials conducted between 1983–1993, suggested that insulin treatment can delay the onset and progression of diabetic complications in about 54% of the patients [13]. However, once poor glycemia occurs, subsequent normoglycemia will not prevent progression towards DR, suggesting that hyperglycemia-induced chronic cellular changes are difficult to reverse [14].

In recent years, the concept of repairing terminally differentiated organs with a cell-based therapy has evolved. The cells used in these approaches are diverse and include tissue-specific endogenous stem cells, endothelial progenitor cells, and bone-marrow derived mesenchymal stem cells. Among cell-based approaches intended to address DR, intravitreal injection of endothelial [15]_ENREF_11 and myeloid progenitor cells [16] have been shown to prevent vascular regression and protect neurons in genetic mouse models of retinal degeneration. Yet to date, the identity and cell surface markers expressed by these cells remain incompletely defined [17]. Further, most of these studies have been performed in non-diabetic models [eg. oxygen induced retinopathy (OIR) and retinal ischemia-reperfusion injury] or genetic models of retinal degeneration that are not diabetic models. Consequently, we and others turned attention to adipose stem cells (ASC), which have functional and phenotypic overlap with pericytes lining microvessels in adipose tissues [18], [19], and which also form robust functional vascular networks *in vivo* by cooperation of ASC with cord blood endothelial cells [20]. Mendel et al recently reported that indeed ASC-derived cells can integrate with retinal vasculature, adapting both pericyte morphology and marker expression, and provide functional vascular protection in multiple murine models of retinal vasculopathy [21]. Although this is a novel observation of direct intravitreal injection compared to the previously described intravenous injection of ASC [22], the use of OIR mice and the Akimba mouse model of DR do not represent true long-term hyperglycemia induced DR models [23]. This prompted us to use the more robust Streptozotocin—induced DR model to test the perivascular integration of ASC to rescue the capillary damage.

Apart from their role as perivascular cells, ASC are also known to produce a variety of angiogenic and antiapoptotic factors [24]. We and others have shown that ASC act in a paracrine manner, as well as by direct physical interaction with endothelial cells, to modulate angiogenesis [25]_ENREF_1, reduce skeletal muscle ischemia and tissue loss [24]_ENREF_3, limit myocardial infarction [26]_ENREF_4, promote skin repair [27], and provide neuroprotective function [28]. These seminal studies have led to the concept that ASC may not only rescue the retina from diabetic capillary damage, but also from neurodegeneration by suppressing inflammation and apoptosis. To test this hypothesis here, we employed a rat model of streptozotocin (STZ)-induced chronic diabetes model to address whether ASC ameliorates not only the structural abnormalities of early DR, but also improves neuronal activity (as assessed by electroretinogram) to enhance visual function. Additionally, we addressed mechanisms by which ASC withstand hyperglycemic stress *in vitro*, and thus may protect retinal endothelial cells *in vivo*. This is an important question, as transplanted cells typically undergo significant cell death, which could hamper the potential benefit of cell transplantation, specifically considering hyperglycemic environment observed in the diabetic vitreous [29].

## Research Design & Methods

### Isolation and characterization of human ASCs

Studies involving human adipose tissue sample collection were approved by Indiana University School of Medicine Institutional Review Board. The adipose specimens were obtained from elective surgical procedures and are deemed normal medical waste products resulting from these procedures. Therefore, collection of de-identified specimens was exempted from informed consent requirements. Human subcutaneous adipose tissue samples obtained from lipoaspiration procedure were processed to isolate ASC as described previously [25]. In brief, the fat tissue was digested in collagenase type I solution (Worthington Biochemical, Lakewood, NJ) under agitation for 1 hour at 37°C and centrifuged at 300 *g* for 8 minutes to separate the stromal cell fraction (pellet) from adipocytes. The pellet was re-suspended in DMEM/F12 containing 10% FBS (Hyclone, Thermofisher.com) filtered through 250 µm Nitex filters (Sefar America Inc, Depew, NY) and centrifuged at 300 *g* for 8 minutes. The cell pellet was treated with red cell lysis buffer (154 mmol/L NH_4_Cl, 10 mmol/L KHCO_3_, 0.1 mmol/L EDTA) for 10 minutes. The final pellet was suspended in EBM-2/5% FBS or in EGM2-MV (Cambrex, East Rutherford, NJ).

ASC cultured for 2 days on culture plastic were harvested with 2 mmol/L EDTA/PBS and routinely checked for both pericyte and mesenchymal cell surface markers including CD10+/CD13+/CD31−/CD34−/CD44+/CD45−/CD73+/CD90+/CD105+ [30].

ASC were labeled with lentiviral GFP per standard procedures. Briefly, ASC were plated at 10000 cells/cm^2^ in EGM-2-MV and three hours later the media on ASC was exchanged with fresh media supplemented with CSCGW-EGFP lentiviral stock solution (pCSCGW-EGFP construct provided by Dr Ken Cornetta, Indiana University Vector Production Facility) and 8 µg/ml of polybrene (Sigma-Aldrich, St Louis, MO). The next day the media on the cells was replaced with fresh culture media, cells were expanded, and sorted for the ones that demonstrated strong expression of fluorescent proteins using FACS Aria Sorter (BD biosciences, Franklin Lakes, NJ). Intact ASC were used during sorting procedure to establish cutoff for autofluorescence. As an additional method, ASC were also labeled with DiI per manufacturers protocol (Vybrant® DiI Cell-Labeling Solution, Life technologies, Carlsbad, CA).

### Diabetic rat model and intravitreal injections

All animal studies were approved by IACUC, Indiana University School of Medicine as per the ARVO Statement for the Use of Animals in Ophthalmic and Vision Research. Athymic nude rats (Hsd:RH-Foxn1^rnu^) were obtained from Harlan Laboratories (Indianapolis, IN) at 5–6 weeks of age. Animals were maintained in a specific pathogen-free environment in positive pressure rooms with a standard 12 hour day/12 hour night cycle. All animals were fed a normal pellet chow (Harlan Teklad, Madison, WI). After acclimatization, diabetes was induced by a single intra-peritoneal injection of a freshly prepared solution of STZ in citrate buffer (pH 4.5) at 55 mg/kg of body weight. Diabetes was confirmed within two weeks with blood glucose (Bayer Contour test strips, Bayer HealthCare LLC, Pittsburgh, PA) levels higher than 350 mg/dL on two consecutive days. Intraperitoneal glucose tolerance tests (GTT) and morphometric assessment of pancreatic islet β-cell mass were performed as previously described [31], [32]. Body weight was measured regularly. Insulin was given (0–3 times per week, 0–2 units SC of NPH insulin, Eli Lilly, Indianapolis) to achieve weight maintenance without preventing hyperglycemia (>250 but <450 mg/dL) and glucosuria. Thus, diabetic rats were insulin deficient but not grossly catabolic. Two months after diabetes onset, rats were anesthetized with isoflurane and intravitreal injections (50,000 to 250,000 of GFP-labeled ASC in 2 µL saline, typically right eye) were performed with a 30-gauge microsyringe (Hamilton, Reno, NV), using a temporal approach, 2 mm posterior and parallel to the limbus. The left eye received an equal volume of saline and served as control.

### Quantitation of retinal pathology

Two months post diabetes induction, rats were euthanized and enucleated eyes were preserved in 10% neutral buffered formalin. The retinal vasculature was isolated from formalin-fixed eyes using the trypsin digest technique as described by us previously [33]. After drying the purified vessel network onto a glass slide, the preparations were stained with hematoxylin and periodic acid Schiff. Acellular capillaries were quantitated in 4–7 field areas in the mid-retina (200× magnification) in a masked manner. Acellular capillaries were identified as capillary-sized vessel tubes having no nuclei anywhere along their length, and were reported per square millimeter of retinal area. Pericyte ghosts were estimated from the prevalence of protruding bumps in the capillary basement membranes from which pericytes had disappeared [33]. At least 1,000 capillary cells (endothelial cells and pericytes) in 5 field areas in the mid-retina (400× magnification) in a masked manner were examined. Ghosts on any already acellular vessel were excluded.

### Albumin Extravasation Assay

Vascular permeability in athymic nude rats was assessed by the albumin extravasation assay method as published previously [34]. Briefly, athymic nude rats after two months post diabetes induction or age matched normal rats were anesthetized and received tail vein injection of FITC-BSA (100 mg/Kg body weight, Sigma-Aldrich) One hour after injection rats were euthanized, perfused with 4% paraformaldehyde, eyes were enucleated and embedded in Tissue-Tek CRYO-OCT Compound (Thermo Fisher scientific, Inc). Frozen sections (5 µm) were cut throughout the retina to obtain 7 sections per animal with 30 µm apart. Using an epifluorescence microscope, extravasation of FITC-BSA from retinal vessels was captured and quantified using Image J software (NIH.gov). The fluorescence values were then normalized to the plasma level of FITC determined by fluorometer (Molecular Devices, Sunnyvale, CA).

### Realtime RT-qPCR analysis of mRNA expression

Seven days after ASC or saline injection, rats were euthanized; enucleated eyes and retinas were collected free of vitreous and flash frozen for mRNA analysis. Each individual retina's were processed for total RNA using NucleoSpin RNA II Kit (Clontech, Mountain View, CA). About 50 ng of RNA was mixed with SYBR green mix containing rat gene specific primers (see Table S1) as per the manufacturer's instructions (iScript One-Step RT-PCR Kit with SYBR, Bio-Rad, Hercules, CA). Samples were analyzed on Applied Biosystems StepOne™ Real-Time PCR (Applied Biosystems, Life technologies, Carlsbad, CA) in a total reaction volume of 20 µL. The thermal cycling program consisted of an initial 10 minutes cDNA synthesis at 50°C followed by 15 minutes Thermo-start activation. The amplification included denaturation at 95°C, followed by 40 cycles of denaturation at 95°C for 15 seconds, annealing at 58°C for 30 seconds, and melting curve analysis. To compare the levels of rat DR gene transcripts between the diabetic saline treated v/s diabetic ASC treated, we used comparative C_P_ method for relative quantification as described previously [35] and expressed compared to saline treated non-diabetic rats. The amount of target gene transcript, normalized to the elongation factor alpha (EF1α)endogenous housekeeping gene transcript and relative to the calibrator, was computed by 2^−ΔΔCP^, where ^ΔΔ^C_P_ = ΔC_P_ (unknown target gene) − ΔC_P_ (calibrator), and ΔC_P_ of target or calibrator is the C_P_ of the target gene subtracted from the C_P_ of the housekeeping gene.

### TUNEL assay for apoptosis

Frozen rat retinal sections were assessed for DNA strand breaks by TUNEL assay as per the manufacturer's instructions (ApopTag Plus In Situ Apoptosis Fluorescein Detection Kit, EMD Millipore, Billerica, MA). In some cases where FITC-BSA or GFP cells were injected, a modified HRP detection system with anti-digoxigenin HRP (1∶500; Roche, Indianapolis, IN) was adopted. In each experiment, adjacent sections incubated without TdT served as control. The total number of TUNEL-positive cells in diabetic rats was normalized to total nuclear cells using MetaMorph analysis (Molecular Devices, Sunnyvale, CA) and shown as a percentage of non-diabetic animals that received saline injection.

### Immunohistochemical analysis

Frozen sections of retina (5–10 µm) were fixed in 2% paraformaldehyde for 10 minutes. For blocking non-specific background staining tissues were exposed to serum free Protein Block (Dako, Carpinteria, CA) for 45 minutes followed by incubation for overnight at 4°C with the anti-alpha smooth muscle actin antibody (αSMA, Clone 1A4, mouse IgG, AbCam, Cambridge, MA; 1∶200), anti-histone human IgG (Clone: AE-4; monoclonal mouse antibody, AbCam; 1∶400) and anti-Von Willebrand factor (vWF, Rabbit Polyclonal Ab, AbCam, 1∶200) antibodies. This was followed by washing and incubation with secondary antibody (Alexa Fluor® 647 goat anti-mouse IgG and Alexa Fluor® 546 rabbit anti-rabbit IgG, Life Technologies at 1∶1000). Retinal tissues without exposure to the primary antibody were used as controls for immunostaining. Stained tissues were counterstained with DAPI and mounted using fluorescent mounting media (Sigma) and visualized using a Nikon Eclipse 80i upright digital microscope (Nikon Instruments Inc., Melville, NY).

### Retinal wholemounts and confocal microscopy

Rats were euthanized by CO_2_ inhalation, and the eyes were enucleated and fixed with 2% paraformaldehyde. Retinas were dissected and set in 24-well cell culture plates. After washing, retinal tissues were blocked with serum free Protein block (Dako) and permeabilized with 0.3% Triton X-100 in PBS at room temperature for 1 to 2hrs. Samples were then incubated overnight in the dark at 4°C with different combinations of antibodies. Vasculature was labeled with either Alexa Fluor® 488 conjugated Isolectin GS-IB_4_ from *Griffonia simplicifolia* (Life Technologies) or rabbit polyclonal collagen IV antibody (Abcam). ASC were identified by GFP or labeled with human IgG antibody (Life Technologies). Pericytes were labeled with mouse monoclonal alpha smooth muscle actin antibody [1A4]. After washing, tissues were incubated with Alexa Fluor® 647 donkey anti-mouse IgG and Alexa Fluor® 546 conjugated goat anti-rabbit IgG antibodies for 3–4 hrs in the dark at 4°C. Retinal tissues were then counterstained with nuclear DAPI and were flat mounted with vitreous side up (Fluorescent mounting media, Sigma) on clean glass slides. Retinal flat mounts were examined under a confocal scanning laser microscope (Olympus FV1000-MPE, Center Valley, PA) configured to eliminate autofluorescence and spectral overlap allowing precise discrimination between the fluorochromes imaged. Z-stacks of confocal images of retinal wholemounts were reconstructed and analyzed (Olympus Fluoview 3.0 software).

### Electroretinography

To assess retinal function in DR model, we performed dark adapted image guided flash focal-electroretinogram (ERG, Micron III, Phoenix Research Labs, Pleasanton, CA) intensity response series in anesthetized rats before and after intravitreal injections of ASC as described previously with slight modifications [36]. Briefly, rats after 2 hr of dark adaptation anesthetized with ketamine and xylazine cocktail. Pupils were dilated with 1% tropicamide and ERG was performed on each rat sequentially beginning with the weakest excitation gradually increasing intensity. At least two regions (nasal and temporal) of the retina were targeted using the deep red real-time retinal image from Micron III camera as a guide. The reference needle was placed between two eyes and the grounding probe was inserted into the base of the tail. Corneal electrode attached to the Micron III camera lens was used to record ERG data. Roughly about 250 µm diameter of the retina was targeted with a 6-ms pulse to obtain twenty traces of readings at different light intensities beginning at 1.1 cd-second/m^2^ that doubled in intensity until reaching 1.5×10^26^ cd-second/m^2^. Using Labscribe2 version 2.34 (iWorx Systems Inc, Dover, NH) the amplitude (implicit time) of the b-wave was measured from the trough to the peak of the first visible b-wave.

### Co-culture of retinal endothelial cells and ASC

For vascular network formation (VNF) assay, Human Retinal Microvascular Endothelial Cells (HREC; ACBRI 181, Cell Systems Corporation, Kirkland, WA) and ASC were co-cultured according to the protocol published previously [37]. Mixture of 10,000 of HREC and 60,000 of ASC (per cm^2^) were re-suspended and cultured in EBM-2/5% FBS medium for 6 days with media exchange at day 3. At the end of the experiment, vascular networks were visualized by staining the cultures with biotinylated Ulex Europaeus Agglutinin I (Vector labs, Burlingame, CA) and anti-αSMA IgG as previously described [37]. On an average of 9 images of each well were captured with 4× objective and processed for total tube length by Angiogenesis Tube formation assay module of MetaMorph software (Moleculardevices.com). Human cord blood derived endothelial cells served as a positive control cell type for HREC as described by us previously [37].

In contact independent co-cultures, HREC (60,000 cells) were plated on the lower surface of 24-well Transwell plates, and ASC (60,000) were plated in Transwell 0.4-µm-pore inserts. After adherence of cells to the designated surfaces, inserts with ASC were moved into the wells containing HREC. Co-cultures were cultivated under EBM-2/0.1% FBS for 3 days with medium change daily with varying doses of glucose (5.5 mM, 25 mM and 55 mM) or mannitol as an osmolality control. At the end of day 3, cells from the bottom were analyzed for cell viability/apoptosis.

### Assessment of Cell viability

*In vitro* ASC viability upon exposure to high glucose were assessed by proliferation and apoptosis assays. Proliferation was assessed by Cell Proliferation Assay kit based on the cleavage of the tetrazolium salt WST-1 to formazan by cellular mitochondrial dehydrogenases (EMD Millipore). Briefly, about 10000 ASC were plated in a well of a 96-well flat bottom plates and left for 2 hours for attachment. Following this, the medium was removed and 200 µl of the varying doses of glucose & mannitol were distributed into each well and incubated for upto 72hrs at 37°C, 5% CO_2_ incubator. After incubation, 10 µL of the WST-1 dye working solution were added to the wells and plates were incubated for further 4 hours. The absorbance (*A*) values of each well were read at 460 nm using an automatic microplate reader (Flexstation, Molecular devices). The percentage viability was calculated using the background-corrected absorbance as follows: % viability  =  [(*A* of experimental well)/*A* of control well] *100.

As an additional method for cell viability, apoptotic levels in ASC were measured after exposure to increased doses of glucose and mannitol as described above. After 24 hours, cells were fixed in a glyoxal based formalin-free fixative (Prefer Ready-to-use, Anatech Ltd, Battle Creek, MI) for 20 minutes and washed with PBS. Subsequently, cells were immunostained with active caspase-3 antibody (affinity-purified rabbit polyclonal antibody, Promega, Madison, WI; 1∶500) and detected with an Alexa Fluor 546 conjugated rabbit antibody. The total fluorescent intensity in a given well was computed as a ratio of total nuclear DAPI positive cells and assessed percent apoptotic rate in cells treated with glucose compared to normal glucose control. As a positive control to induce apoptosis, staurosporine (Cell Signaling, Danvers, MA) at 1 µM was used in both the experiments while normal human dermal fibroblast (HDF) cells were used a negative cell type for ASC which also originates from stroma of mesynchyme.

### Statistical Analysis

Data analysis from *in vivo* are expressed as mean ± SEM of a group of n≥6–8. *In vitro* cell culture experimental data is shown as mean ± SD of triplicate measurements and repeated independently three additional times. Statistical significance was determined by Student's *t* test or one way ANOVA using GraphPad Prism software (La Jolla, CA). A probability value p<0.05 was considered statistically significant.

## Results

### Development of diabetes in athymic nude rats is accompanied by features of early stage retinopathy

**Figure 1:**
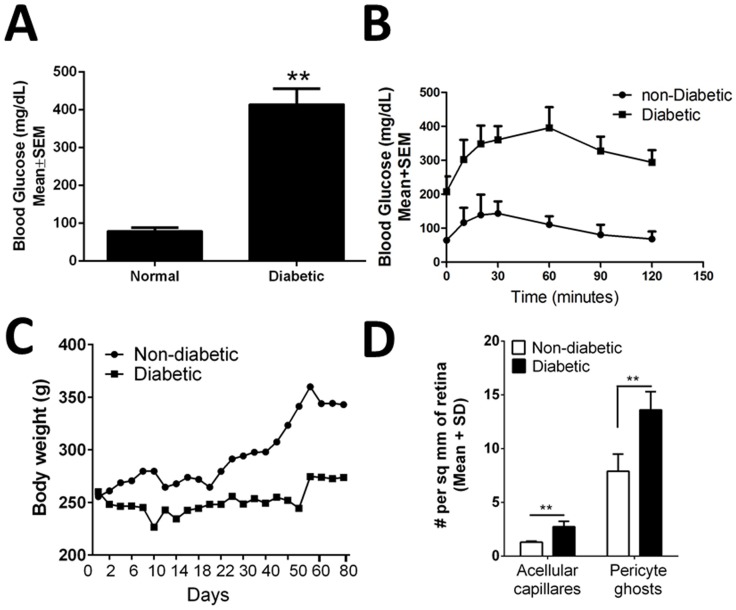
Athymic nude rat developed diabetes with early DR features. (A) Athymic nude rats developed sustained hyperglycemia compared to non-diabetic controls. Blood glucose level was elevated as early as week one and remained high until after two months. n = 6 per group, **p<0.01. (B). Two months post diabetes induction, intraperitoneal GTT was performed. As expected STZ-induced (square boxes) rats developed impaired glucose tolerance compared to non-diabetic controls (circles). Data shown is an average of n = 6 per group. (C). Body weights over a period of 2 months measured on a regular basis demonstrated a progressive increase in body weight in non-diabetic animals, while the diabetic animals failed to gain the weight. Data shown is an average of n = 8–12 per group. (D). Two months post diabetes induction a significant (**p<0.01) increase in acellular capillaries and pericyte ghosts in diabetic nude rats observed compared with non-diabetic age matched rats. Data is from n = 14–16 per group.

Athymic nude rats (6 weeks old) treated with 55 mg/kg of STZ developed sustained hyperglycemia when compared to non-diabetic control rats. While blood glucose levels in non-diabetic animals were 97±15 mg/dL, diabetic animals had significantly elevated levels (413±55 mg/dL, p<0.01) as early as two days to a week after STZ, and remained >250 mg/dL until after two months of STZ injections (Fig 1a). Approximately two months post diabetes induction and prior to ASC injections, GTT was performed to document glucose intolerance and results were compared to age-matched non-diabetic rats. As expected STZ-treated (Fig 1b, squares) rats developed impaired glucose tolerance compared to non-diabetic controls (Fig 1b, round). Fasted STZ rats given 1 g/kg body weight of D-Glucose demonstrated elevated blood glucose levels of >250 mg/dL throughout the period compared to non-diabetic rats which cleared the glucose load within the two hours. Consistent with this, pancreatic islet β cell area in diabetic rats was significantly decreased (p<0.05) compared to non-diabetic rats (Figure S1 in File S1). Diabetic rats in this study were hyperglycemic and failed to gain weight at a normal rate compared to age matched non-diabetic controls (Fig 1c). Taken together, it is suggestive that these athymic nude rats develop diabetes when induced with STZ. Interestingly, intravitreal injection of ASC had no effect on elevated random blood glucose level after 3 weeks post transplantation, but demonstrated a slight natural expected increase in body weights in these rats (Figure S2 in File S1).

Currently, a nude rat model of DR that display early stage retinopathy that can be used to test stem cell therapies is not available [23]. In this regard, we tested if the athymic nude rat will develop DR features. Two months post STZ injection, these rats developed substantial increase in vascular leakage, apoptosis, inflammation (see below) and a modest but statistically significant increase in acellular capillaries (2.75±0.5, diabetic rats v/s 1.3±0.1 non-diabetic rats acellular capillaries/sq. mm retina; p<0.01) and pericyte ghosts (13.6±1.7, diabetic rats v/s 7.9±1.6 non-diabetic rats acellular capillaries/sq. mm retina; p<0.01) in the retina confirming the development of early stage DR model (Fig. 1D and Figure S3 in File S1).

### Intravitreal injection of ASC improves retinal function in the diabetic athymic nude rat

**Figure 2:**
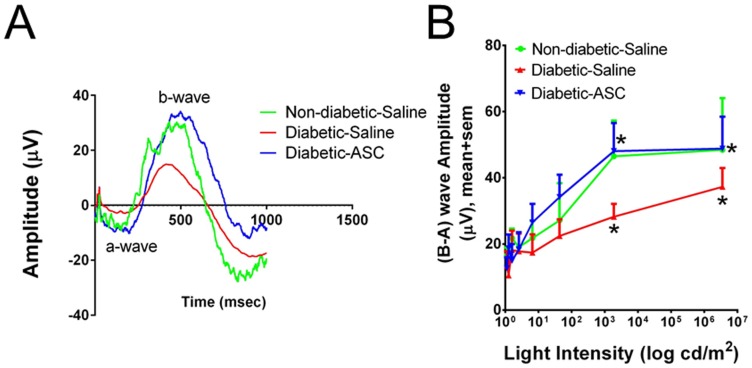
Intravitreal injection of ASC improves retinal function in the diabetic athymic nude rat. Two months post diabetes induction ERG was recorded in anesthetized rats at day 6 with saline and ASC injections and compared with non-diabetic rats that received saline injections. A representative ERG waves from dim flash to bright flash over time is computed (A). Notable differences in b-wave observed in diabetic animals compared to non-diabetic group, which was near completely normalized with ASC. The b-wave amplitudes computed against different light intensities (B) revealed a significant (*p<0.05) decrease in amplitude at high intensities in diabetic rats that received saline compared to non-diabetic rats that received saline. Interestingly, the decrease in amplitude is significantly (*p<0.05) alleviated with ASC at day 6 compared to diabetic rats that received saline. The data shown is from a group size of n = 6–8 animals.

Prior to the development of any anatomically visible retinal changes, diabetic eyes displayed a significant decrease in neuronal function, as measured by ERG. As shown in Fig. 2, focal ERG revealed a decreased response of the “b” wave in diabetic eyes that received saline. However, a single intravitreal injection of ASC restored near normal ERG response within 7 days and remained high for 3 weeks suggesting possible restoration of vision in this model (Fig 2 and Figure S4 in File S1). Interestingly, at day 7 diabetic rats that received saline demonstrated a significant decrease in ERG response compared to same animals on day 0, while the diabetic rats that received ASC clearly demonstrated a significant increase in ERG response. We have tested a dose response of ASC in a different set of animals ranging from 50,000 to 250,000 cells per eye. Since we did not find any major significant differences in ERG response, for consistency in subsequent experiments we have used 250,000 cells per eye.

### Intravitreal injection of ASC alleviate vascular leakage in diabetic athymic nude rat

**Figure 3:**
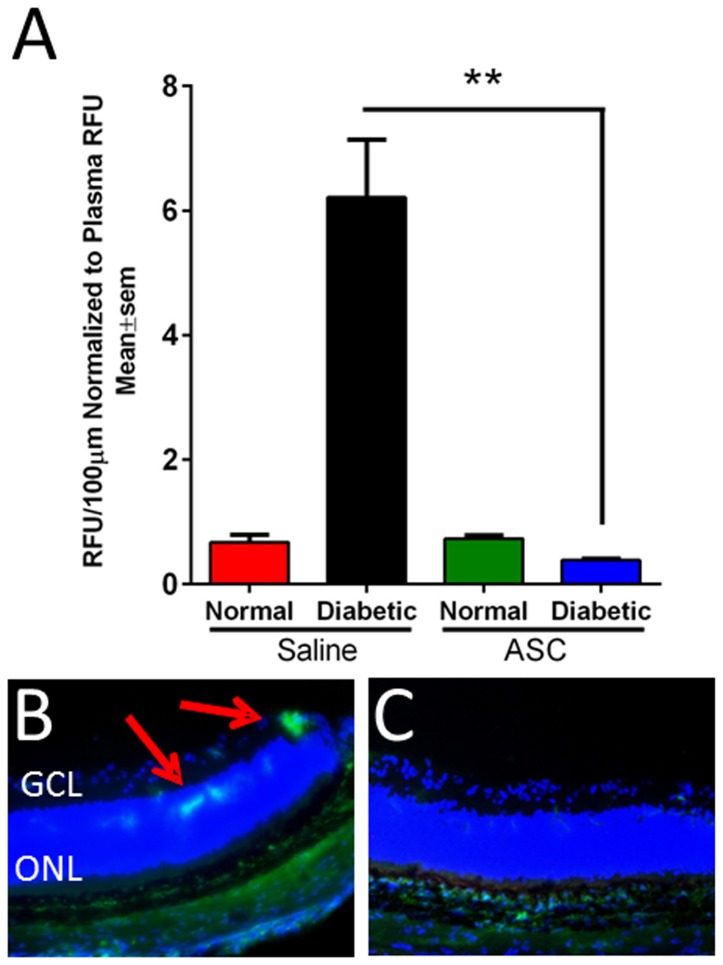
Increase in vascular permeability in diabetic athymic nude rats is alleviated with intravitreal ASC injection. (A) Two months post diabetes induction or age matched normal rats were injected intravitreally with saline (n = 8–10/group) or ASC (n = 6–8/group; 250,000 cells in 2 µL of saline). At day 7, rats were injected with 100 mg/kg of FITC-BSA via tail vein. After 1hr, rats were euthanized and perfused with 4% paraformaldehyde, and eyes enucleated and embedded. About ten 10 µm sections of equal area were analyzed for FITC fluorescence and normalized to total plasma fluorescence. A significant (**p<0.01) increase in leakage in diabetic rats was alleviated by ASC injection. Representative immunofluorescence image of a diabetic retina received saline (B) and diabetic retina treated with ASC (C) showing leakage (20× original magnifications). RFU = Relative fluorescence units.

Vascular leakage has been shown to be a characteristic feature of early stage DR [3]. Athymic nude rats (two months post diabetes induction) and age matched normal rats were injected with saline or ASC. At day 7, vascular leakage was assessed by FITC fluorescence in retinal sections as normalized to total plasma fluorescence (Fig 3). The increase in leakage was dramatic and significant in diabetic animals that received saline compared to non-diabetic animals (6±1 relative fluorescence units, RFU/100 µm v/s 1±1 RFU/100 µm, n = 6; p<0.01). Furthermore, a single intravitreal injection of ASC reduced the FITC leakage significantly compared to diabetic animals that received saline injection (0.5±0.2 RFU/100 µm v/s 6±1 RFU/100 µm, n = 8; p<0.01). As an additional confirmation of this data, in representative diabetic rats, fluorescein angiography (FA) was performed to assess the leaky vessels. FA performed after intraperitoneal injection of fluorescein sodium in live rats revealed the characteristic pattern of vascular leakage in diabetic rats that was completely absent from age matched non-diabetic controls (Figure S5 in File S1).

### Intravitreal injections of ASC alleviate apoptosis in the diabetic athymic nude rat

**Figure 4:**
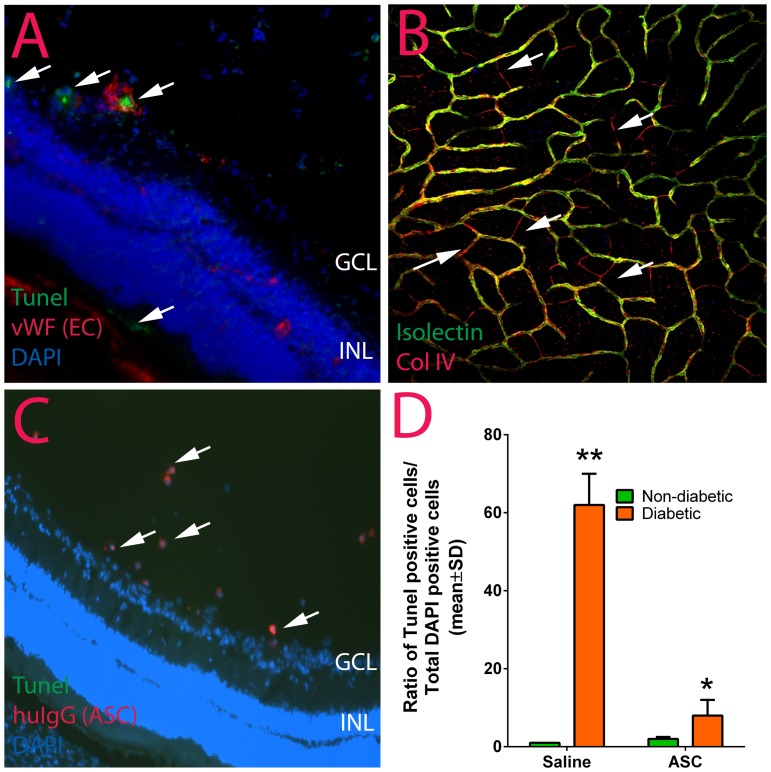
Increase in apoptosis in diabetic athymic nude rats is diminished with intravitreal ASC injection. Two months post diabetes induction or age matched normal rats were assessed for apoptosis by TUNEL and detected by confocal immunofluorescence. (A) diabetic rats that received saline demonstrated extensive TUNEL positive cells in ganglion cell layer (GCL) and photoreceptor layer (arrows). (B) Increased apoptosis correlated with increase in acellular capillaries (red Collagen IV only, arrows) in retinal wholemounts stained with Collagen IV and isolectin (yellow colocalization). (C) Human IgG positive ASC could be located within the vitreous and the retinas were not positive for TUNEL suggesting a decrease in apoptosis. (D) Quantification of TUNEL positive cells in normal and diabetic rats that received saline or ASC treatment. The number of TUNEL positive cells in ASC treated diabetic rats was significantly lower compared to the number of positive cells in diabetic rats that received saline. Data are representative of n = 6–8 per group. **p<0.01 diabetic-saline v/s non-diabetic-saline; *p<0.05 diabetic-saline v/s diabetic-ASC.

To investigate the therapeutic role of ASC in retinal cell death in early DR, we assessed apoptosis by TUNEL assay. Two months after induction of diabetes, the number of TUNEL positive cells in saline treated diabetic retina increased significantly compared to age matched non-diabetic controls. The TUNEL positive cells were mainly located in clusters, which were found proximal to the capillaries and ganglion cell layer (Fig 4). Of particular note, these TUNEL positive cells were also positive for vWF suggesting apoptosis in endothelial cells corresponding with increased acellular capillaries. The total number of TUNEL-positive cells normalized to total nuclear cells and expressed as a percentage of non-diabetic animals that received saline injection demonstrated a significant increase in diabetic animals (62±8%, diabetic v/s 1±0%, non-diabetic, p<0.024). Furthermore, a single intravitreal injection of ASC into diabetic animals at day 7 resulted in a 54% reduction in TUNEL-positive cells (8±4%, n = 9; p<0.024) compared to diabetic animals with saline treatment. In nondiabetic animals, there was no significant change in the number of TUNEL-positive cells after treatment with ASC (2±0.5%, n = 6; p>0.05).

### Intravitreal injection of ASC alleviates inflammation in the diabetic athymic nude rat

**Figure 5:**
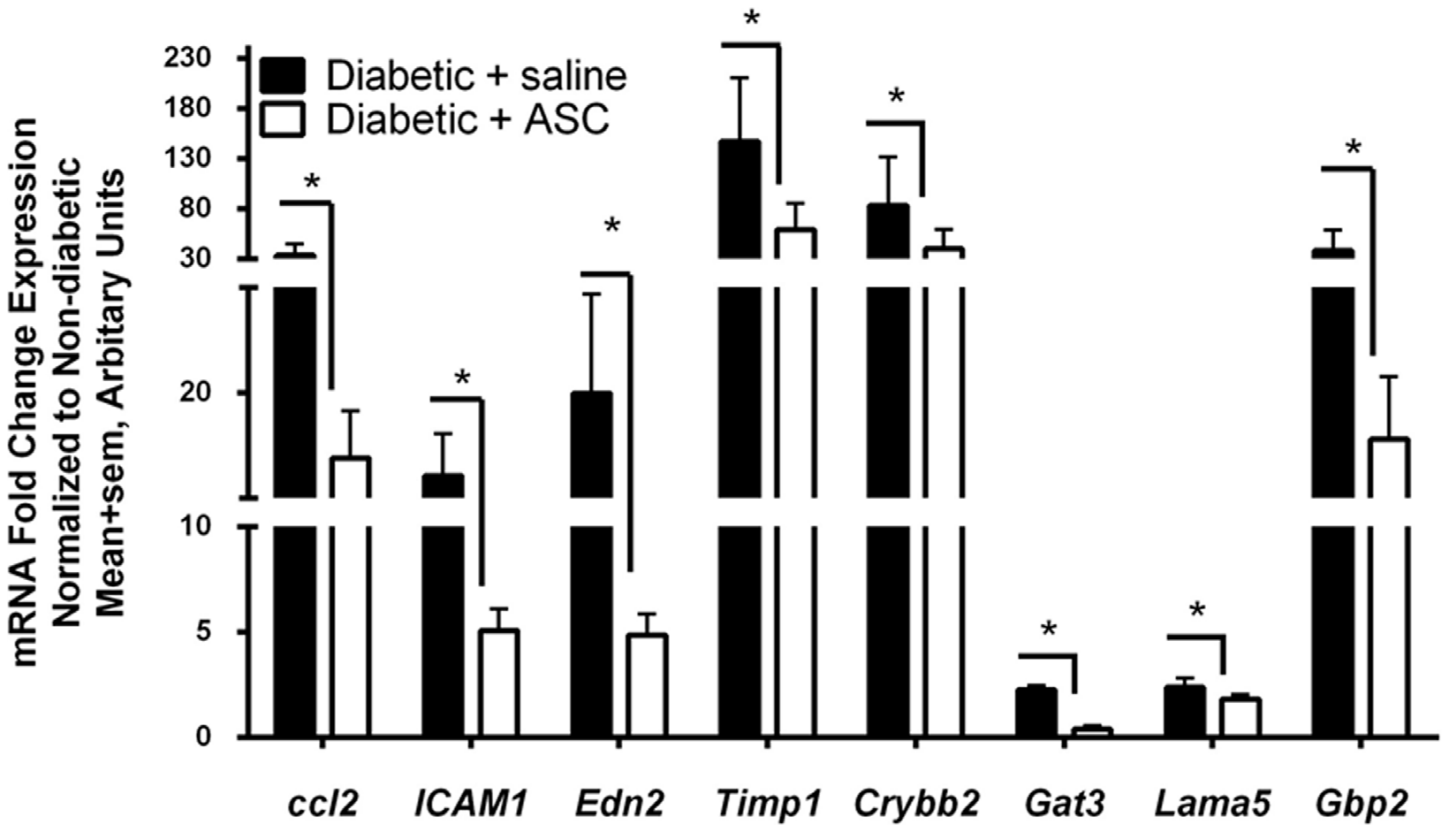
Intravitreal ASC injection decreased DR related gene expression in diabetic athymic rats. Two months post diabetes induction intravitreal injections of ASC at day 7 were assessed for DR related gene expression changes. Using rat gene specific primers, total RNA was subjected to single step RT-qPCR and results expressed normalized to non-diabetic rats that received saline injection. Note a significant increase in genes implicated in DR in diabetic athymic nude rats that received saline. A single intravitreal injection of ASC at day 7 significantly downregulated these genes compared to diabetic retina. Data are from n = 6–8 rats per group. *p<0.05.

To investigate the effect of ASC in retinal inflammation as observed in early DR, we assessed mRNA gene transcripts by quantitative real-time RT-qPCR assay. Several pro-inflammatory cytokines and biomarker panel genes implicated in DR research [38], namely ccl2, ICAM-1, Edn2, Timp1, Crybb2, Gat3, Lama5 and Gbp2 were significantly upregulated (>2 fold, p<0.01) in diabetic retina that received saline compared to non-diabetic retina (Fig. 5). Interestingly, a single intravitreal injection of ASC at day 7 significantly (p<0.05) downregulated these genes compared to diabetic retina.

### Incorporation of intravitreal ASC into the host vasculature in diabetic athymic rats

**Figure 6:**
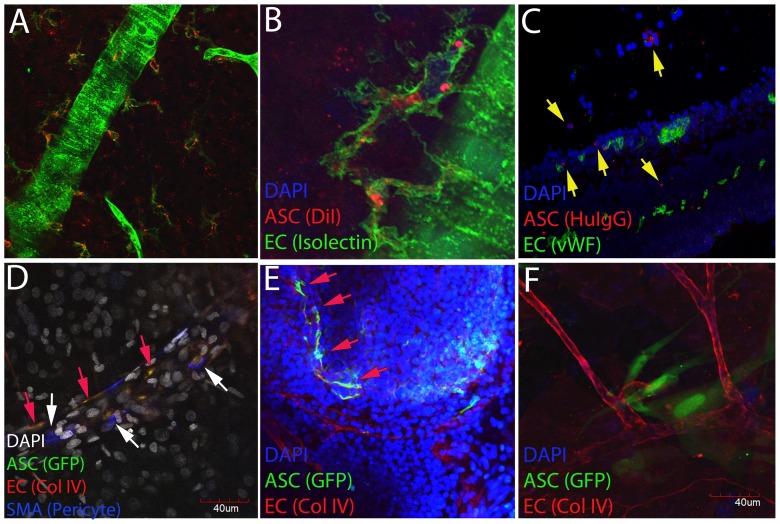
Incorporation of intravitreally delivered ASC into host vasculature in diabetic athymic rats. Two months post diabetes induction or age matched normal rats were intravitreally injected with either saline or ASC (250,000 cells in 2 µL of saline). At day 7 or day 21, rats were euthanized and retinal wholemounts were prepared after various antibodies immunostaining as mentioned in methods. Confocal images of Z-stacks from representative animals demonstrated (A) DiI labeled ASC in close relationship with host vasculature (B) Zoomed from A, (C) co-localization of vWF and human histone IgG within the retina, (D) GFP-ASC co-localized with endothelial Collagen IV (yellow arrows) and pericyte specific αSMA (white arrow heads), (E) GFP-ASC at day 21 assuming perivascular position (yellow arrows) and (F) age-matched non-diabetic rats with GFP-ASC did not co-localize perivascularly with host capillaries.

To investigate the localization and fate of intravitreally injected ASC, retinal wholemounts were prepared and assessed by confocal microscopy. A number of DiI-labeled ASC were found in close relationship to the host vasculature within 7 days after transplantation (Fig. 6A&B). Human ASC could easily be identified by GFP and/or human histone IgG antibody co-localizing with capillaries stained with vWF antibody (Fig. 6C) and/or pericyte marker αSMA (Fig. 6D). In a complementary set of diabetic animals, GFP-labeled ASC were injected and left for 21 days. These GFP cells were similarly found lining the perivascular position of the host capillaries. This localization occurred more frequently in diabetic rats that were assessed at day 21 (Fig. 6E) compared to age-matched non-diabetic rats that received GFP-ASC (Fig. 6F) which failed to take perivascular position.

### ASC can withstand high glucose stress *in vitro* with no effect on cell proliferation and cell survival

**Figure 7:**
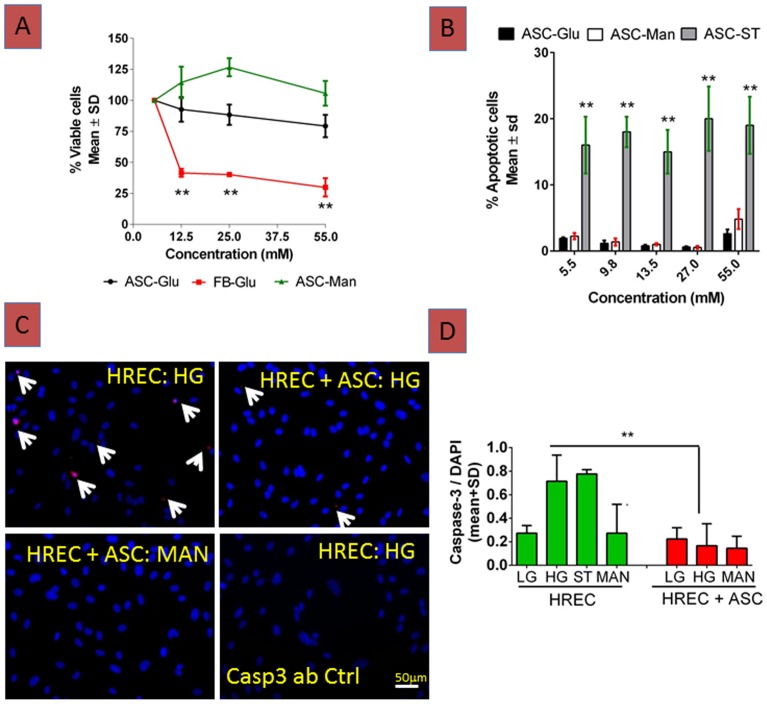
*In vitro* ASC withstand hyperglycemic stress with minimal apoptosis and increase retinal endothelial survival. (A). Number of viable cells at normal (5.5 mM) or high glucose concentrations did not change at 72hrs as evidenced from the quantity of formazan product measured at 490 nm, is directly proportional to the number of living cells in culture. On the other hand, normal human fibroblasts (HDF) demonstrated a significant decrease (**p<0.01) in viable cells in comparison to ASC. Mannitol (Man) used as osmolality control did not affect ASC. (B). ASC treated with increasing doses of glucose or mannitol were fixed in Prefer fixative. Cells in each well were stained using an active caspase-3 antibody (Promega Corp). The % apoptotic cells were calculated based on total fluorescence intensity of caspase-3 stained cells relative to total cell count. As expected staurosporine (ST, 1 µM) caused massive cell death (**p<0.01) at low glucose (5.5 mM), whereas both high glucose and mannitol did not show any significant (p>0.05) apoptosis compared to low glucose. (C). HREC co-cultured with ASC and exposed to high glucose (HG; 25 mM) were protected from apoptosis as shown by decrease in active caspase-3 staining in co-cultures compared to HREC cultured alone with HG (arrows). (D). Quantification of total fluorescence intensity of caspase-3 stained cells relative to total cell count by MetaMorph show a significant decrease in caspase-3 staining, **p<0.01. Mannitol (MAN) at 25 mM served as an osmolality control and had no effect.

To investigate the effect of high glucose stress on ASC proliferation, an MTT assay was performed with varying doses of glucose. Mannitol was used as an osmolality control. The total percentage of viable cells after 72hrs remained constant at 80±10% throughout the varying doses of glucose, and demonstrated only a marginal decrease at 55 mM (Fig. 7A). In contrast, HDF demonstrated a decreased ability to proliferate with 30±10% of viable cells. Effects of high glucose on cell viability and the beneficial effect of ASC were also confirmed by caspase-3 staining for apoptosis. As expected, high glucose did not induce caspase-3 activation in ASC, while staurosporine, a known proapoptotic agent that activates caspase-3, increased its level by 5 fold (Fig. 7B).

### ASC protect HREC from high glucose induced stress and form vascular networks *in vitro*

To investigate the effect of high glucose on HREC viability contact independent co-cultures were performed and caspase-3 activity was assessed. As expected HREC cultured under high glucose demonstrated a robust increase in caspase-3 staining compared to normal glucose treated HREC [39], [40] or osmolality control mannitol (Fig.7C&D). On the other hand, HREC co-cultured with ASC alleviated high glucose stress induced apoptosis suggesting ASC protect HREC under hyperglycemic conditions.

**Figure 8:**
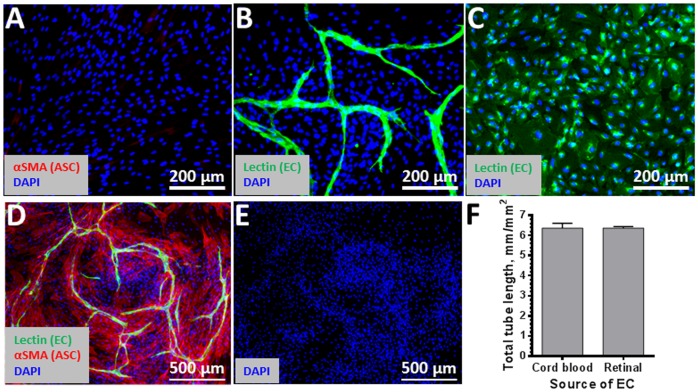
Retinal endothelial angiogenic network formation in the presence of ASC. Retinal endothelial cells formed robust capillary like structures (green cord like structures) when co-cultured with ASC by day 6 of incubation (B, D and E). In addition, the networks were stabilized by co-cultured ASC and immunostained positively for alpha smooth muscle actin (D) producing an elevated density of cells near the networks (E). Both ASC (A) and HREC cultured alone (C) demonstrated no such networks. Of note, the vascular network formation by HREC is comparable to those of cord blood derived endothelial cells previously described [37] (F). Ulex Lectin staining is shown in green, alpha smooth muscle actin–in red, nuclei dye DAPI - in blue.

Previously we have shown that ASC forms robust vascular networks with other endothelial cells [37]. Here we assessed the vascular network formation by HREC when in co-culture with ASC in the absence of any exogenous extracellular matrix proteins or growth factors. While ASC or HREC alone did not form vascular networks as expected (Fig. 8A & C), HREC cultured with ASC spontaneously rearranged into vascular-like cord structures on the top of an ASC monolayer (Fig 8B). Formation of vascular networks by HREC was accompanied by ASC migration and accumulation in direct proximity to HREC cords, producing an elevated density of cells near the networks (based on DAPI staining, Fig 8E) and reduction in density between cords. Additionally, co-culture resulted in a significant increase in αSMA expression in ASC and its organization into fibers, selectively in those ASC that were in direct contact or proximity with HREC (Fig 8D), while those ASC cultured alone demonstrated limited αSMA expression (Fig 8A). Of note, the vascular network formation by HREC is comparable to those of cord blood derived endothelial cells previously described [37] (Fig. 8F).

## Discussion

This study provides a comprehensive demonstration establishing intravitreal injection of ASC as a therapeutic cell type that can play both a reparative and a protective role in the early stages of DR. It also supports the understanding that the beneficial effects of ASC involve the downregulation of hyperglycemic stress induced apoptosis, vessel stabilization and improvement in neuronal activity. Moreover, this study characterizes a novel and robust athymic nude rat model that develops early stages of DR, and which can be used to address transplantation studies with various human stem cells.

ASC are multipotential mesenchymal progenitor cells that we and others have recently demonstrated to have functional and phenotypic overlap with pericytes closely encircling microvessels in multiple human organs including adipose tissue [18], [41], [42]. Importantly, these cells have been shown to have a direct role in providing microvascular support as pericytes *in vitro* [37], *ex vivo* [43], [44] and *in vivo* [20], [45], [46]. Because ASC are an abundant and easily isolated population of adult stem cells from cosmetic lipoaspirations [47], they are an excellent source for future pericyte replacement in DR as well as in other chronic wound therapies. To this end, evidence suggests that ASC have pericyte-like properties, secrete proangiogenic molecules, maintain vascularization in inflamed tissue [48], and aid diabetic wound healing [49], [50]. Direct evidence that ASC play a therapeutic role in DR came from a study in which intravenously administered ASC in the STZ induced DR rat model demonstrated an improvement of BRB integrity, with few donor cells differentiated into photoreceptor or astrocytes-like cells [22]. However, it is not clear whether the observed beneficial effect in BRB breakdown was secondary to the lowering of hyperglycemia in this model, and not to a direct effect of donor ASC in the damaged retina. A related study employed intravitreal injection of TGF-β1 treated ASC, which were shown to differentiate into pericytes could integrate into the retinal vasculature in OIR model, and rescued BRB breakdown in the Akimba DR model in the absence of chronic hyperglycemia [21]. Because the neovascular changes observed in the Akimba mouse are not due to long-term hyperglycemia, as in human DR [23], we developed a STZ induced chronic hyperglycemia DR model. We here demonstrate for the first time that intravitreal injection of ASC in this model not only decreases BRB breakdown, but also are found paired with host vasculature and adjacent to capillaries, possibly suggesting pericyte replacement within 3 weeks of cell transplantation. Although more studies are warranted, the ability to provide such perivascular cells in the early stages of disease would represent a significant advancement in our understanding of the role of ASC cell therapy in DR.

DR develops as sustained metabolic dysregulation inflicts progressive damage to the retinal microvasculature. This increases vascular permeability, and, in advanced stages, leads to the aberrant proliferation of vascular endothelial cells [51]. Importantly, diabetic retinal vascular leakage, capillary non-perfusion, and endothelial cell damage are temporally and spatially associated with retinal leukocyte stasis in early experimental diabetes. Changes in endothelial cells, including the expression of ICAM-1, an important component of leukocyte recruitment, have been suggested to play a role in endothelial activation and permeability. In our model, we show a significant increase in ICAM-1 expression, concurrent with other relevant inflammatory cytokines implicated in development of DR, suggesting a similar pattern in our model. Interestingly, a decrease in ICAM-1 levels was observed at day 7 following provision of ASC. More studies are needed to link the downregulation of ICAM-1 to improvement in this model. However, the observation of a significant decrease in vascular permeability complementing these transcriptional data in our model *in vivo* suggests that ASC produced factors may affect the tight junctions of endothelium and thus may rescue retinal endothelium from damage and loss of vascular permeability.

Complementary to the promotion of vessel stability, ASC-mediated trophic effects on the neurovascular system[28], [52] may prevent or delay the onset of DR. Consistent with previous studies, visual deficits in the early-stage DR in rat models have been correlated with retinal dysfunction even before vascular dysfunction. Similarly, here we demonstrate significant changes in ‘b’ wave amplitudes represented by bipolar and Muller cells in our diabetic rats within two months of diabetes. These early ERG changes are in support of the idea that neuronal and Müller cell dysfunction occurs at the same time in streptozotocin-induced hyperglycemia[53]. Although we were unable to trace oscillatory potentials, which were highly correlated with DR progression [54], our data with intravitreal injection of ASC demonstrating the restoration of near normal ‘b’ wave amplitudes suggested a beneficial role for ASC in DR. The intracellular signaling pathways that mediate this effect on b-wave needs further study. Both indirect trophic factor effects of ASC on neural retina, and a direct differentiation of ASC into pericytes to preserve host vasculature, may play roles in this apparently therapeutic outcome.

Extensive literature suggests that athymic nude rats are suitable for use in transplantation, tumor therapy, and carcinogenesis research, among many other purposes [55]–[59]. We have developed the model of chronic Type I diabetes in these outbred rats in order to better study the function of ASC [60]. Diabetic rats in this study were hyperglycemic, and failed to gain weight at a normal rate compared to age matched non-diabetic controls. The ASC injected diabetic rats were comparably hyperglycemic with diabetic rats injected with saline. The blood glucose levels in these two groups for the entire duration of the experiment were similarly elevated compared to normal non-diabetic rats, suggesting that intravitreal ASC or saline did not cause any changes in blood glucose levels [61]. Consistent with these random blood glucose data, the intraperitoneal GTT in diabetic rats demonstrated poor glucose tolerance, and decreased islet β-cell mass compared with non-diabetic rats. These observations were also complemented by a substantial increase in vascular leakage, apoptosis and inflammation. It is interesting to note that although these rats are T cell-deficient, they display normal ranges of counts for granulocytes, monocytes/macrophages, erythrocytes, B cells and natural killer (NK) cells in the blood [62]. In addition, these rats have normal levels of proinflamatory cytokines such as interferon-γ tumor necrosis factor-α [62]. With increased age, nude rats develop T-like cells expressing CD3 and T-cell receptor (TCR). Though their phenotype in peripheral tissues resembles that of normal T cells, consisting mainly of CD4 or CD8 cells, they lack alloreactivity *in vivo* and their TCR repertoire is more of an oligoclonal nature [63]. Therefore, it is predictable that nude rats may develop DR, owing to the normal level of leukocytes and proinflamatory molecules [64], [65]. This varies from other models, such as NOD SCID mice, which failed to develop DR features [66], possibly because they are deficient in T, B lymphocyte and NK cells [67]. Since T cell-like cells in our athymic nude rat may fail to recognize the allogeneic cells (eg. stem cells), ours may be an excellent DR model that can be used to test xenogeneic stem cell transplants. Consistent with this concept, observations involving ASC up to three weeks in these rats did not show any immune rejection features (data not shown).

Previous studies performed in STZ-diabetic rodent models demonstrated significant physiological and biochemical changes within 1–2 months. However, development of acellular capillaries and loss of pericytes occurred only after 6 months, and continued to increase until 18 months [33], [68]. Surprisingly, our athymic nude rat model demonstrated a modest but statistically significant increase in acellular capillaries and pericyte ghosts in the retina after only two months of diabetes, confirming the accelerated development of early stage DR model. Further long-term studies are needed to ascertain if the trend will continue to increase. Contrary to our expectations, the acellular capillary and pericyte ghost analysis with ASC at seven days post-transplantation resulted in further increase in these parameters (data not shown). It is possible that ASC will require a longer duration to ameliorate additional pathological features, highlighting; temporal analyses of the effects will be a subject of future studies.

Our observation of early DR features is also supported by a recent biomarker panel consisting of 14 genes were suggested to be altered in DR pharmacotherapeutic research [38]. We identified several genes to be upregulated (at least 2 fold or greater) in our rat model. Interestingly, many of these genes were modulated with a single intravitreal injection of ASC, suggesting transcriptional control in gene expression may be a beneficial role for ASC in DR. Specifically, genes coding for proteins involved in the immune response (gbp2) and in intracellular signal transduction pathways activated by cytokines and chemokines, such as adhesion (lama5), inflammation (ccl2; edn2) and transcription (stat3), were strongly represented. This indicates a critical role of ASC in the early modulation of the immune response and neural retinal rescue. For example, in the beginning of DR, the level of ccl2 begins to increase, and continues to increase during the development of the disease. It is primarily secreted by retinal neurons, and plays an important role in retinal microglial activation, which may be an important clue in the pathogenesis of DR [69]. Another target that is decreased with ASC injection is an inflammatory factor, endothelin 2 (Edn2), which promotes central nervous system remyelination [70] and acts as a macrophage attractant [71], both of which are characteristic of retinal disease [72], [73]. Future studies with more detailed temporal gene expression analysis and the specific proteins involved (gain and/or loss of function experiments) are needed.

It is imperative that for treatment of diabetic vessel damage, transplanted cells should be equipped with a cellular and molecular armamentarium capable of withstanding the *in vivo* diabetic microenvironment [29], [74]. To this end, we show that ASC withstand hyperglycemic stress with sustained cell viability, and relatively low levels of apoptosis despite increasing concentrations of glucose. In addition, ASC markedly enhanced HREC survival under hyperglycemic conditions; and in contact co-cultures, ASC formed robust vascular networks with HREC, much as with cord blood endothelial cells in previous studies[37]. Although the mechanisms are unclear, we speculate that paracrine trophic factors released by ASC play key roles by both stabilizing vasculature, and in protecting retinal cells from diabetic damage. In favor of this hypothesis, we have recently identified physiologically-relevant levels of several anti-apoptotic, anti-inflammatory and chemotactic proteins (such as tumor necrosis factor-inducible gene 6, TSG6; stanniocalcin 1, STC-1; Rantes, CCL5; Stem cell factor, SCF; [18] & unpublished data Jie Xie and Keith March, 2013) in ASC conditioned media, which have been shown to mediate some of the beneficial effects of mesenchymal stem cells [75], [76].

One of the caveats to ASC transplantation therapy for DR is the secretion of angiogenic growth factors produced by ASC, such as VEGF and HGF [24]. Because excessive growth factors have been linked to proliferative DR, a careful regulation of ASC differentiation may be necessary. However, depending on the microenvironment, mesenchymal stem cells have been shown to produce paracrine trophic factors that may modulate between a pro-angiogenic to anti-angiogenic environment, potentially supporting pathological disease [77]–[80]. Based on this evidence, future studies with both ASC and the trophic factors from ASC are needed in both early DR and late stage proliferative DR models. In addition, it will be helpful to determine whether there are any long-term ocular and systemic side effects of intravitreal injections of ASC in DR [81]. Finally, better understanding is needed of the cell signaling mechanisms that play key roles in neuro-vascular repair with ASC.

In conclusion, using a newly generated model of an athymic nude rat that develops early stage DR, we have shown that a single intravitreal injection of ASC substantially limited the development of diabetic ocular complications. *In vitro*, ASC displayed sustained proliferation and decreased apoptosis when subjected to hyperglycemic stress, and enhanced retinal endothelial vascular network formation, supporting the notion that ASC are appropriate for cell transplantation studies in the diabetic environment. By evaluating this approach in rodent models, we will be in a better position to determine how such an approach can be best translated to human clinical trials. These diabetic rat studies provide a foundation for the design of our future clinical studies in human diabetic retinopathy.

## Supporting Information

File S1 **Combined file of supporting figures and tables.** Figure S1: Decreased pancreatic β-cell mass in diabetic athymic nude rat. (A) Pancreata from STZ treated diabetic rats and non-diabetic controls were sectioned and stained for insulin (red) and counterstained with hematoxylin (blue). Original magnification, ×100. β cell mass in diabetic rats was quantitated by MetaMorph software. Note a significant decrease in β cell mass in diabetic rats compared to non-diabetic controls (*p<0.05). Data shown is a representative of n = 5 per group. Figure S2: Body weights unchanged in the diabetic athymic nude rat with intravitreal injection of ASC. Two months post diabetes induction intravitreal ASC injections were performed. Body weight was measured daily for the next three weeks. Insulin was given periodically. Intravitreal injection of ASC had no effect on elevated blood glucose after 3 weeks post transplantation but demonstrated a slight natural expected increase in body weights in these rats. The data shown is from a group size of n = 6–8 animals. Figure S3: Retinal trypsin digests reveal acellular capillaries and pericyte ghosts. Two months post diabetes induction trypsin digests were performed as described in methods. Acellular capillaries (red arrows) were identified as capillary-sized vessel tubes having no nuclei anywhere along their length. Pericyte ghosts (black arrow) were estimated from the prevalence of protruding ―bumps in the capillary basement membranes from which pericytes had disappeared. At least 1,000 capillary cells (endothelial cells and pericytes) in 5 field areas in the mid-retina (400× magnifications) in a masked manner were examined for quantification. Data is a representative photomicrograph from n = 6–8 per group. Figure S4: Long term improvement in the retinal function in the diabetic athymic nude rat with intravitreal injection of ASC. Two months post diabetes induction ERG was recorded in anesthetized rats at day 0 (green line) and performed intravitreal injections of either saline (left) or ASC (right). At day 7 and day 21 post ASC injections, ERG was measured. A representative ERG waves from dim flash to bright flash over time is computed (A). Typical b-wave amplitudes plotted against time clearly demonstrated a decreased in amplitudes with saline at day-7 (red line; left) while animals that received ASC (right), clearly had an increase. This increase in amplitudes measured on day-21 (blue line) remained high in ASC group suggesting a long lasting effect of ASC treatment in diabetic retinal function. The data shown is from a group size of n = 6–8 animals. Figure S5: Increased retinal vascular permeability in diabetic athymic nude rat. Fluorescein angiography (FA) was performed to assess the leaky vessels (Micron III retinal imaging system, Phoenix Research Labs) based on standard procedures. Sodium fluorescein (0.05 ml of 25%) injected through tail vein was captured at the same time frame between diabetic and non-diabetic rats clearly revealed a significant leakage of fluorescein. In addition, fundus examination of live anesthetized diabetic and non-diabetic rats using bright field imaging revealed hemorrhages in diabetic rats that were near completely absent in non-diabetic rats. Data shown is a representative of n = 3–6 per group. Table S1: Realtime RT-qPCR primer pairs. Rat gene specific primers were designed using Primer3, a widely used program for designing PCR primers available at http://www-genome.wi.mit.edu/genome_software/other/primer3.html.
(PPTX)
